# Selection of housekeeping genes for gene expression studies in larvae from flatfish using real-time PCR

**DOI:** 10.1186/1471-2199-9-28

**Published:** 2008-03-06

**Authors:** Carlos Infante, Makoto P Matsuoka, Esther Asensio, José Pedro Cañavate, Michael Reith, Manuel Manchado

**Affiliations:** 1 IFAPA Centro *El Toruño*, CICE, Junta de Andalucía, Camino Tiro de pichón s/n, 11500 El Puerto de Santa María, Cádiz, Spain; 2 Institute for Marine Biosciences, National Research Council, 1411 Oxford Street, Halifax, Nova Scotia, B3H 3Z1, Canada

## Abstract

**Background:**

Flatfish metamorphosis involves major physiological and morphological changes. Due to its importance in aquaculture and as a model for developmental studies, some gene expression studies have focused on the understanding of this process using quantitative real-time PCR (qRT-PCR) technique. Therefore, adequate reference genes for accurate normalization are required.

**Results:**

The stability of 12 potential reference genes was examined during larval development in Senegalese sole (*Solea senegalensis*) and Atlantic halibut (*Hippoglossus hippoglossus*) to determine the most suitable genes for qRT-PCR analysis. Transcription levels of genes encoding β-Actin (ACTB), glyceraldehyde-3P-dehydrogenase (GAPDH), annexin A2 (ANXA2), glutathione S-transferase (GST), ornithine decarboxylase (ODC), hypoxanthine phosphoribosyltransferase (HPRT1), ubiquitin (UBQ), elongation factor 1 alpha (eEF1A1), 18S ribosomal RNA, and the ribosomal proteins S4 (RPS4) and L13a (RPL13a) were quantitated. Two paralogous genes for ACTB were analyzed in each of both flatfish species. In addition, two paralogous genes for GAPDH were studied in Senegalese sole. RPL13a represented non-orthologous genes between both flatfish species. GeNorm and NormFinder analyses for expression stability revealed RPS4, UBQ and eEF1A1 as the most stable genes in Senegalese sole, Atlantic halibut and in a combined analysis. In all cases, paralogous genes exhibited differences in expression stability.

**Conclusion:**

This work suggests RPS4, UBQ, and eEF1A1 genes as useful reference genes for accurate normalization in qRT-PCR studies in Senegalese sole and Atlantic halibut larvae. The congruent results between both species in spite of the drastic differences in larval development suggest that selected housekeeping genes (HKGs) could be useful in other flatfish species. However, the finding of paralogous gene copies differentially expressed during development in some HKGs underscores the necessity to identify orthologous genes.

## Background

**Figure 1:**
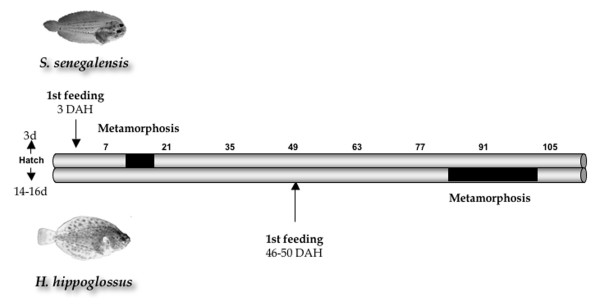
**Larval development scheme in Senegalese sole and Atlantic halibut.** Scale is indicated in days after hatching (DAH). Metamorphosis period is indicated in black.

Pleuronectiformes (flatfish) are a broad taxonomic group comprising 11 families and about 500 species worldwide, and some of them are of high commercial interest both in fisheries and aquaculture. Senegalese sole, *Solea senegalensis*, and Atlantic halibut, *Hippoglossus hippoglossus*, are two representative species of Pleuronectidae and Soleidae families, respectively. Pleuronectidae (right-eye flounders) and Soleidae (soles) families include 93 species in 39 genera and 89 species in 22 genera, respectively [1,2]. All these species share in common an asymmetrical body development and a bottom-dwelling mode of life. Although they possess bilateral body symmetry during the larval stages, they undergo drastic morphological and physiological changes in order to become an asymmetric benthic juvenile. Senegalese sole and Atlantic halibut show clear differences in the timing of larval development and metamorphosis (Figure 1). In Atlantic halibut, hatching occurs approximately two weeks after fertilization. The yolk-sack period extends to 46–50 days after hatching (DAH) and the metamorphosis begins very late with the migration of the left eye about 80 DAH, taking 2–3 weeks [3]. In contrast, Senegalese sole hatches 3 days after fertilization and first feeding occurs at 3 DAH. Metamorphosis takes only one week between 12 and 19 DAH [4]. In spite of the differences in the timing of metamorphosis, this process is mediated by thyroid hormones (THs) in both species [5,6]. Because of the intrinsically interesting role in development as well as the subsequent effects on characteristics such as growth, deformities or malpigmentations, which are important in aquaculture, flatfish metamorphosis has been the subject of gene expression studies using PCR-based methods [7-9]. However, adequate and reliable reference genes for accurate quantification remain to be validated.

Quantitative real-time PCR has become one of the most widespread techniques for mRNA gene expression analysis due to its accuracy, broad dynamic range, sensitivity and reproducibility [10-12]. Although absolute and relative quantitation approaches are possible, the latter is preferred due to the normalization of sample variations using internal housekeeping genes (HKGs). These HKGs act as endogenous controls that allow for the correction of experimental variations caused by pipetting errors, inhibitory compounds, reverse transcription (RT) efficiency or quality of starting material [11]. Traditionally, highly expressed genes such as glyceraldehyde-3-phosphate dehydrogenase (GAPDH), β-actin (ACTB) or 18S ribosomal RNA have been considered as putative HKGs. Due to their key role in metabolism, cytoskeleton and ribosome structure, respectively, it is generally assumed that they are expressed at a constant level in different tissues, cells or experimental treatments. However, there is increasing evidence that their expression can vary during development or in response to external treatments [13-15]. In addition, some of them (i.e. GAPDH and other glycolytic enzymes as well as ribosomal proteins) possess functional paralogous genes that exhibit tissue-specific expression patterns (muscle, liver and/or brain) [16-19]. As an alternative, the use of multiple internal control genes have been proposed [20]. Different algorithms have been developed to choose the most appropriate HKG combination set [20-22]. However, candidate reference genes need to be validated under the specific experimental conditions being evaluated.

The development of large-scale genomics of Senegalese sole and Atlantic halibut (Pleurogene project; [23]) has made possible the availability of a large number of EST sequences. We have identified commonly used reference genes in both species. Twelve candidate HKGs for gene expression analysis during larval developmental in Senegalese sole and Atlantic halibut have been investigated. The possible use of the selected HKGs for gene expression studies in other flatfish species is discussed.

## Results

### mRNA transcription levels of potential HKGs during larval development

A total of 12 genes were evaluated as potential HKGs in Senegalese sole and Atlantic halibut. Owing to the widespread use of GAPDH and ACTB as reference genes, different paralogous genes identified in Senegalese sole and Atlantic halibut were included in the analysis. Only ACTB1 and GAPDH1 were considered orthologs in Senegalese sole and Atlantic halibut as determined by phylogenetic analysis and gene expression profile in tissues. In addition, RPL13a genes were paralogous copies between the two flatfish species. ODC could not be detected in Senegalese sole EST libraries and thus it was only analyzed in Atlantic halibut.

**Figure 2:**
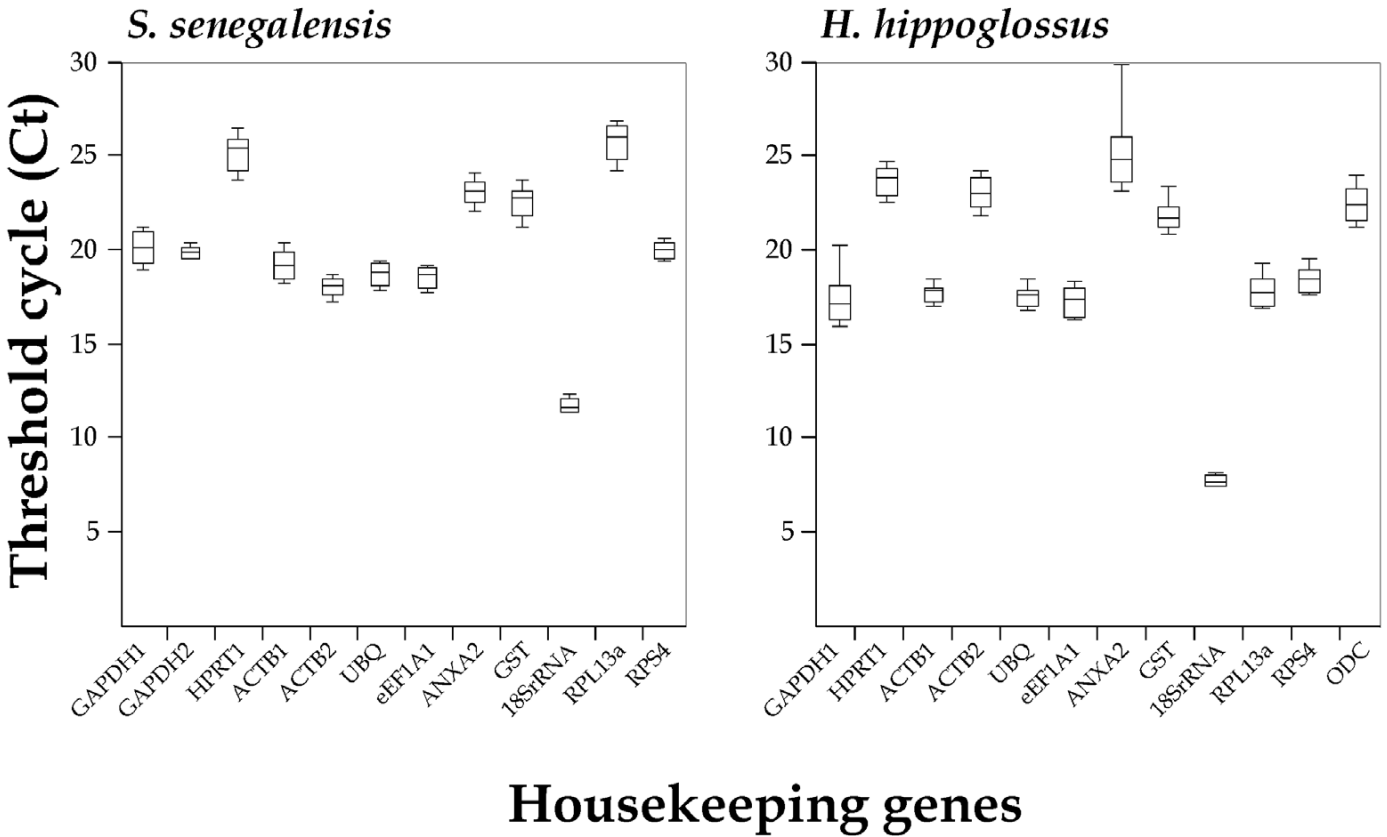
**Transcriptional levels of candidate reference genes (Ct values) over all larval stages.** Bars indicate the 25/75 percentiles and the line marks the median.

The Ct value indicates the fractional cycle at which the fluorescence intensity equals the threshold fluorescence. Ct values are inversely related with the abundance of a specific transcript in the sample and they have to be converted to a linear form by using the amplification efficiency to estimate gene expression changes. Ct values of HKGs in Senegalese sole ranged between 11.05 (18S rRNA) and 27.20 (RPL13a) (Figure 2). In Atlantic halibut, Ct values varied between 7.35 (18S rRNA) and 30.16 (ANXA2). HPRT1, ANXA2 and GST were expressed at low levels in both flatfish species (median Ct values above 20 cycles). The smallest Ct variation was exhibited by GAPDH2 (1.25) and 18S rRNA (1.80) in the Senegalese sole and by 18S rRNA (1.25), ACTB1 (2.45) and HPRT1 (2.61) in the halibut. On the contrary, RPL13a (4.40), GAPDH1 (3.45) and ACTB1 (3.45) showed the most variable expression levels in the sole, whereas ANXA2 (7.34) and GAPDH1 (5.99) were the most variable in the halibut.

**Figure 3:**
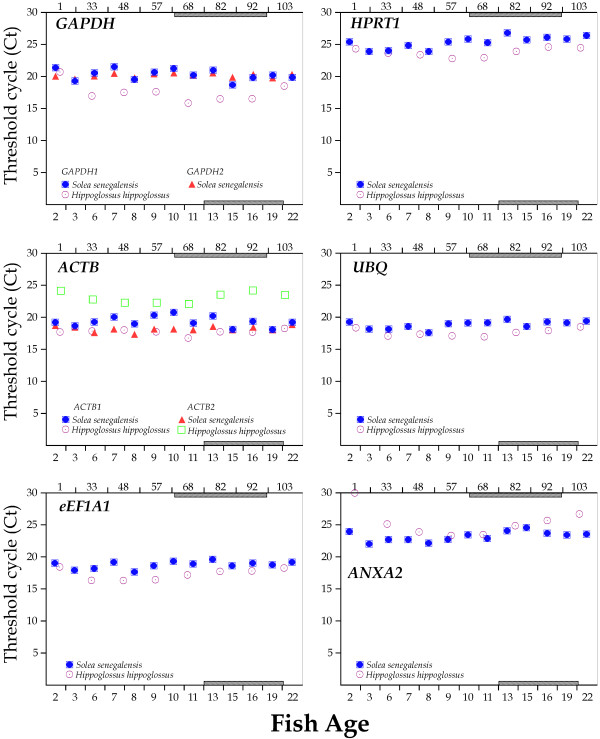
**Transcriptional levels (Ct values) of candidate reference genes (GAPDH, HPRT1, ACTB, UBQ, eEF1A1, ANX2) during larval development.** Age is indicated as days after hatching (lower, Senegalese sole; upper, for Atlantic halibut). Metamorphosis period is shaded.

**Figure 4:**
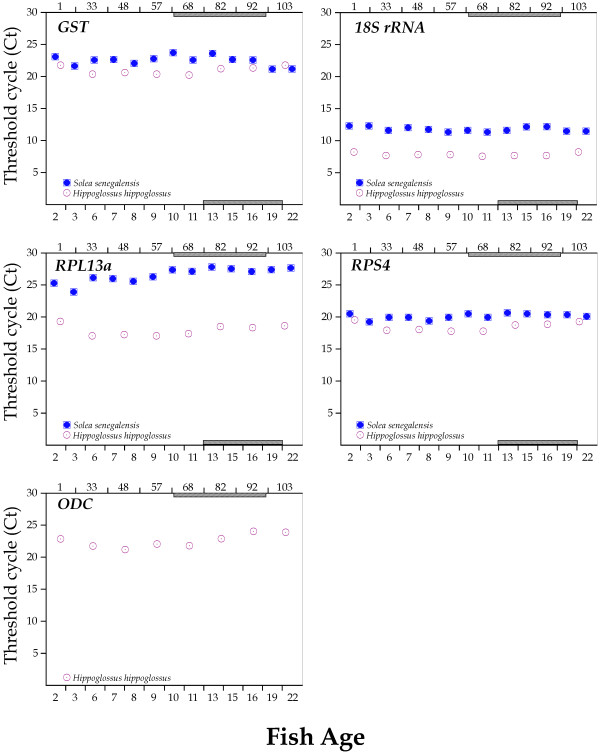
**Transcriptional levels (Ct values) of candidate reference genes (GST, 18S rRNA, RPL13a, RPS4, ODC) during larval development.** Age is indicated as days after hatching (lower, Senegalese sole; upper, for Atlantic halibut). Metamorphosis period is shaded.

The mRNA expression profiles for all HKGs studied in Senegalese sole and Atlantic halibut during larval development are depicted in Figures 3 and 4. ANXA2 exhibited the most pronounced variation in Atlantic halibut since expression levels dropped 6.75 Cts from hatching to 57 DAH. In Senegalese sole, RPL13a showed the highest Ct variation (3.95) between 3 and 13 DAH.

### Gene expression stability analysis

**Figure 5:**
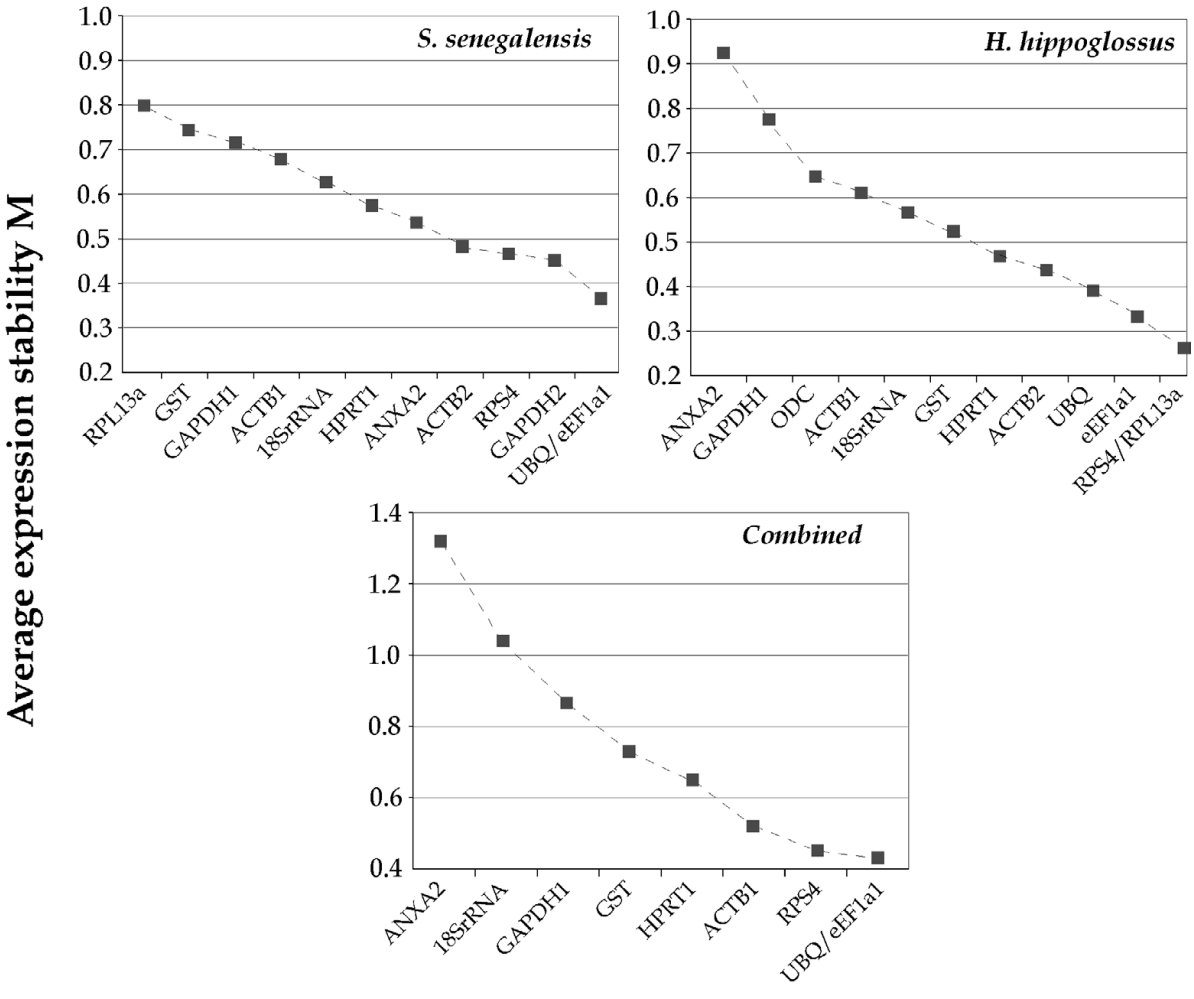
The geNorm ranking of reference genes over larval development in Senegalese sole, Atlantic halibut and combined analysis.

Expression stability for each HKG during larval development in Senegalese sole and Atlantic halibut was analyzed using the geNorm and NormFinder software. GeNorm (or pairwise comparison approach) ranks HKGs according to the similarity of the expression profiles across the sample set. All investigated genes exhibited high average expression stability (M) values (lower than 1 in both species). In the Senegalese sole, ACTB2, RPS4, GAPDH2, UBQ and eEF1A1 were the most stable genes with M values lower than 0.5. In Atlantic halibut, HPRT1, ACTB2, UBQ, eEF1a1, RPS4 and RPL13a exhibited M values lower than 0.5 (Figure 5). Additionally, a combined analysis to determine the most stable genes in both flatfish was carried out. In this analysis, only the putative orthologous genes in both flatfish species were included. Although higher M values were observed, ACTB1, RPS4, UBQ and eEF1A1 showed M values lower than 0.6.

**Figure 6:**
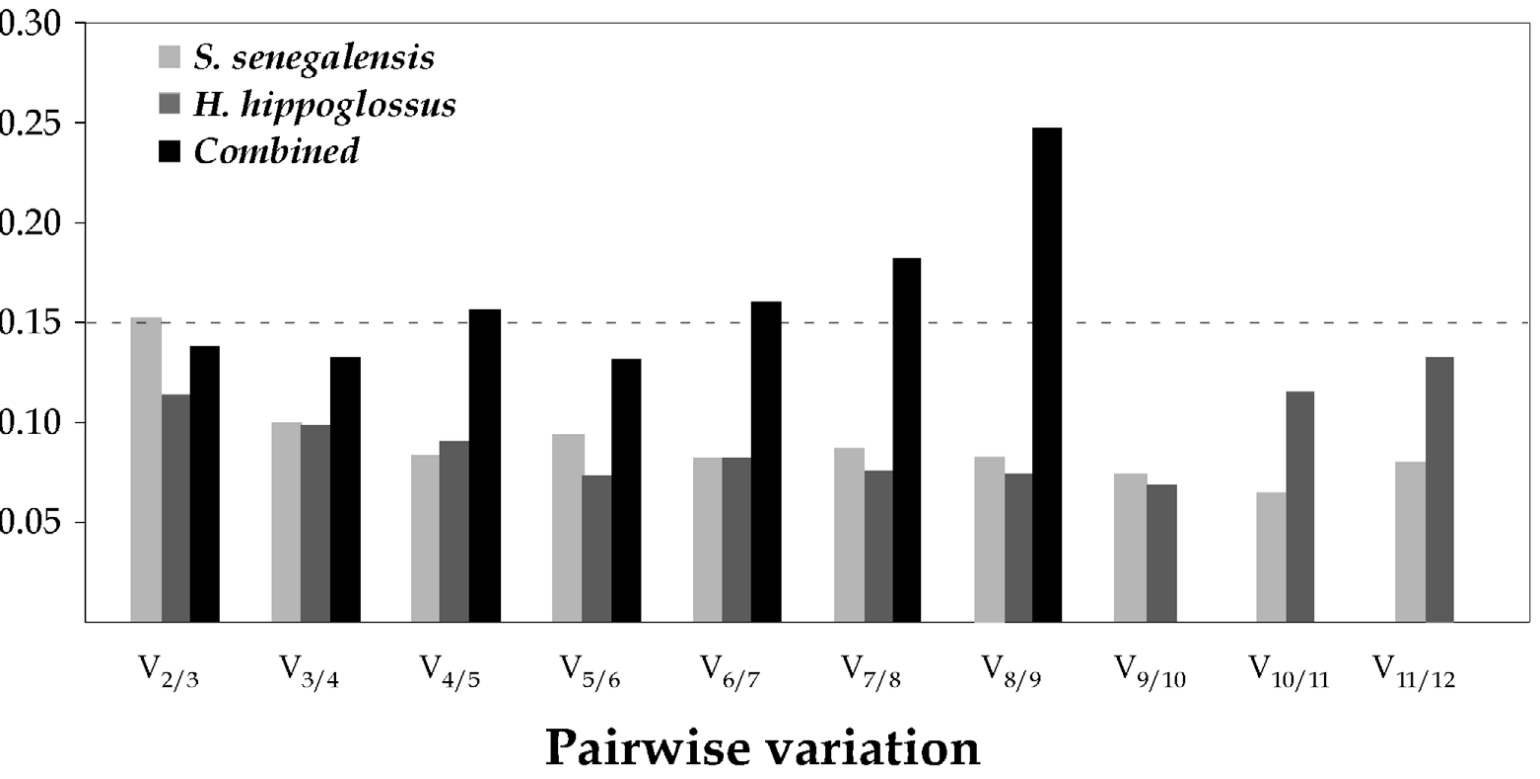
**Optimal number of control genes for normalization as determined by geNorm for Senegalese sole, Atlantic halibut and combined analysis.** Threshold value (0.15) is indicated with a dashed line.

To determine the minimum number of HKGs necessary for an accurate normalization, a pairwise variation V_n/n+1 _analysis was performed (Figure 6). If we take into account the default cut-off value of 0.15 [24], geometric averaging of 3 genes in Senegalese sole and 2 in Atlantic halibut would be required for accurate normalization. Combined analysis yielded a V_2/3 _value of 0.138 although the inclusion of a third reference gene reduced slightly the pairwise variation (V_3/4 _= 0.132).

NormFinder (Model-based approach) ranks the best candidate HKGs according to their minimal combined inter- and intragroup expression variation [21]. Results of NormFinder analysis for our data are shown in Table 1. In Senegalese sole, GAPDH2, RPS4, ACTB2, eEF1A1 and UBQ appeared as the most stable genes (stability between 0.219 and 0.289). In Atlantic halibut, RPS4, UBQ, RPL13a, GST, HPRT1 and eEF1A1 showed the highest stability values (0.159–0.348). These HKG data sets agree with those obtained using geNorm with slight differences in the ranking order. Combined analysis showed RPS4, UBQ and eEF1A1 as the most stable genes (0.278–0.413)

**Table 1:** The ranking of candidate reference genes according to their expression stability by geNorm and NormFinder in Senegalese sole, Atlantic halibut and combined analysis.

Rank	GeNorm			Rank	NormFinder		
	
	*S. senegalensis*	*H. hippoglossus*	*Combined*		*S. senegalensis*	*H. hippoglossus*	*Combined*
			
1/2	UBQ-eEF1A1	RPS4-RPL13a	UBQ-eEF1A1	1	GAPDH2	RPS4	RPS4
3	GAPDH2	eEF1A1	RPS4	2	RPS4	UBQ	UBQ
4	RPS4	UBQ	HPRT1	3	ACTB2	RPL13a	eEFA1
5	ACTB2	ACTB2	ACTB1	4	eEF1A1	GST	GST
6	ANXA2	HPRT1	GST	5	UBQ	HPRT1	HPRT1
7	HPRT1	GST	GAPDH1	6	ANXA2	eEF1A1	ACTB1
8	18S rRNA	18S rRNA	18S rRNA	7	HPRT1	ACTB2	GAPDH1
9	ACTB1	ACTB1	ANXA2	8	GST	18S rRNA	18S rRNA
10	GAPDH1	ODC		9	GAPDH1	ACTB1	ANXA2
11	GST	GAPDH1		10	ACTB1	ODC	
12	RPL13a	ANXA2		11	18S rRNA	GAPDH1	
				12	RPL13a	ANXA2	

## Discussion

Flatfish metamorphosis is characterized by drastic morphological and physiological changes that determine the switch from a pelagic to a benthic lifestyle. During this period, erythrocyte populations and myosin light chains change to adult types [25,26], gastric glands differentiate [27] and an asymmetrical pigmentation develops [8,28]. Owing to the interest of larval stages from flatfish in developmental studies and their impact on essential characteristics of interest in aquaculture (digestion, pigmentation, growth, etc), some gene expression surveys have focused on this developmental period. However, appropriate HKGs that ensure reliable and accurate mRNA quantitation are required. To our knowledge, this is the first study that evaluates the suitability of potential HKGs for gene expression analysis in flatfish larvae. Twelve potential HKGs in the pleuronectid Atlantic halibut and the soleid Senegalese sole were investigated. The high similarity for the most stable HKGs in spite of the differences in the timing of metamorphosis (12–19 DAH in Senegalese sole, and 80–100 DAH in Atlantic halibut) and metamorphic stages (S1–S4 in Senegalese sole [4], and stages 6–9 [29] in Atlantic halibut), gives support and highlight the suitability for gene expression studies in other flatfish.

Two different analytical approaches were used to study the most suitable HKGs during larval development. The pairwise comparison approach (geNorm) selects the most suitable HKGs on the basis of the variation of expression ratios between HKGs expression across sample set. Genes with the lowest M (internal control gene-stability measure) values have the most stable expression [20]. However, the geNorm algorithm is highly dependent on the assumption that none of the genes being analyzed are co-regulated. Although most HKGs analyzed in this study are involved in different cellular functions, such co-regulation cannot be ruled out since some genes of the translational apparatus have been analyzed and the broad effect of THs on metabolism and flatfish metamorphosis [5,30,31]. Hence, HKG expression data were also analyzed using a model-based approach (NormFinder). This methodology ranks candidate genes on the basis of the minimal inter- and intragroup variation [21]. Both experimental approaches identified a similar HKG subset as the most stable. Although only two and three genes were necessary for accurate normalization in Senegalese sole and Atlantic halibut, respectively, and only two in the combined analysis as determined by GeNorm, the inclusion of one additional gene clearly improved the pairwise variation in all cases. Hence, RPS4, UBQ and eEF1A1, involved in protein biosynthesis and degradation, appeared as the most suitable HKG set for accurate normalization in gene expression studies during larval development in flatfish.

Three rounds of large-scale gene duplications have been identified in fish [19,32]. These duplications are responsible, at least in part, for the speciation, the adaptive radiations and the high morphological complexity of fish [33]. The majority of these gene duplicates are lost or silenced during evolution, and only in some instances they can either acquire new functions or divide the ancestral original functions [19,34]. Several paralogous genes including pseudogenes have been described for glycolytic enzymes [18,19], ribosomal proteins [16,35], or elongation factor 1 alpha [14], some of which are routinely considered as potential HKGs. Our data demonstrate that expression profiles and stability vary between paralogous gene copies. GAPDH2 exhibited higher gene stability than GAPDH1 in Senegalese sole. Similarly, ACTB2 showed higher stability than ACTB1 in both species as determined both with geNorm and NormFinder approaches. If we compare the ranking of the two species, the RPL13a paralogous genes were ranked differentially in the two species. In contrast, ACTB1 and GAPDH1 orthologous genes were ranked in a similar way. These data underscore the necessity for a phylogenetic analysis to demonstrate orthology in order to select the appropriate HKG in other flatfish and to make expression profiles comparable between species. In addition, the design of primers is a key point. Due to the high similarity in the coding sequence between some paralogous genes, it becomes necessary to confirm that the paralog of interest is amplified by sequencing of the PCR products or by designing the primers in divergent regions of the UTRs. The simultaneously cross-amplification of different paralogous genes that are differentially expressed would render a consensus expression profile that would make the comparisons among species difficult.

GAPDH has been traditionally considered as a HKG due to its key role in the glycolytic pathway. However, GAPDH is associated with several functions during evolution such as membrane fusion, microtubule bundling, phosphotransferase activity, nuclear RNA export, DNA replication and repair, apoptosis, age-related neurodegenerative disease, prostate cancer and viral pathogenesis (reviewed in [36]). There is increasing evidence that the expression of this gene is modified during development, in tissues and by physiological conditions [13-15]. Our expression data ranked GAPDH1 (muscle-specific isoform) as an inadequate HKG in both Senegalese sole and Atlantic halibut. These data agree with recent findings that have demonstrated a down-regulation of GAPDH1, contrary to GAPDH2, by THs [18], a key regulator of metamorphosis in the two species [5,6]. This differential regulation between both GAPDH paralogous genes would explain the higher expression stability of GAPDH2 (brain-specific isoform), ranked as the best HKG by NormFinder, and the inadequacy of GAPDH1 as HKG during larval development in the two flatfishes.

## Conclusion

Our results indicate that RPS4, eEF1A1 and UBQ genes may be good candidate reference genes for larval development studies in flatfish as selected by geNorm and NormFinder. However, the application of these genes to achieve an accurate normalization in gene expression studies in other species requires the identification of putative orthologous genes.

## Methods

### Source of fish and experimental rearing conditions

All experimental Senegalese sole larvae were obtained from fertilized eggs collected from breeding tanks where breeders spawned naturally under ambient conditions. Eggs were incubated at a density of 2000 eggs L^-1 ^in 300 L cylinder conical tanks with gentle aeration and one water exchange every two hours. Temperature and salinity during all experiments were 20°C and 38 ppt, respectively. Newly hatched larvae were transferred to a 400 L tank at an initial density from 45 to 50 larvae L^-1 ^with a 16L:8D photoperiod and a light intensity of 600–800 lux. Larvae were fed rotifers (*Brachionus plicatilis*) between 3 DAH and 9 DAH. From 7 DAH enriched brine shrimp (*Artemia *sp.) metanauplii were fed until the end of the experiment. Pools of larvae from 2 to 22 DAH (n = 3) were collected, washed with DEPC water, frozen in liquid nitrogen and stored at -80°C until analysis.

Atlantic halibut larval samples were obtained from Scotian Halibut Limited (Clark's Harbour, Nova Scotia, Canada). Fertilized eggs and hatched larvae were incubated at 6 – 7°C until 5 – 6 weeks of age after hatching. After this stage, larvae were raised at 10.0 – 11.2°C. The samples were taken at 1 DAH (hatching), 33, 48, 57, 68, 82, 92, and 103. Sampled larvae were anaesthetized in overdosed tricaine methane sulfonate (TMS, Syndel Laboratories, Vancouver, BC, Canada), and then, transferred to RNAlater (Ambion, Austin, TX, USA) and kept overnight at 4°C. The samples were removed from RNAlater and individually stored in a 1.6 ml microfuge tube at -80°C until RNA was isolated. Ten, 5, and 2 individuals were pooled to isolate total RNA from samples at 1, 33, and 48 DAH, respectively. One whole fish was used to obtain total RNA at other stages. Three RNA samples were obtained for each stage.

### Identification of HKG cDNAs in Senegalese sole and Atlantic halibut

The potential HKGs evaluated in this study were: β-Actin (ACTB), glyceraldehyde-3P-dehydrogenase (GAPDH), annexin A2 (ANXA2), glutathione S-transferase (GST), ornithine decarboxylase (ODC), hypoxanthine phosphoribosyltransferase (HPRT1), ubiquitin (UBQ), elongation factor 1 alpha (eEF1A1), 18S ribosomal RNA (18S rRNA), and the ribosomal proteins S4 (RPS4) and L13a (RPL13a). Expressed sequence tags (ESTs) encoding HKGs from Senegalese sole and Atlantic halibut were identified after sequence analysis of normalized cDNA libraries constructed from different larval stages and adult tissues. ODC was only identified in *H. hippoglossus*. Two different genes encoding for GAPDH (referred to as GAPDH1 and GAPDH2) [18] and for ACTB (referred to as ACTB1 and ACTB2) were identified in both species. Similarly, two RPL13a genes were identified in Senegalese sole. RPL13a-like1 was orthologous of RPL13a gene in halibut. However, to compare gene expression stability between paralogs, the RPL13a-like2 (referred to as RPL13a in the text), probably a pseudogene, was analyzed in this study. Sequences for each HKG in both flatfish species were aligned to assess orthology. GAPDH1 and ACTB1 sequences were orthologs in Senegalese sole and Atlantic halibut. In contrast, ACTB2 and RPL13a were paralogs between both flatfish species. GAPDH2 only was studied in Senegalese sole. Accession numbers and main function of each HKG evaluated are indicated in Table 2.

**Table 2:** Names and Genbank accession numbers of Senegalese sole and Atlantic halibut candidate normalization genes. Putative function is also indicated.

Symbol	Gene Name	Function	Genbank accession number	
			
			*S. senegalensis*	*H. hippoglossus*
ACTB1	Actin, beta	Cytoskeletal structural protein	AB360593	EB029476
ACTB2*	Actin, beta	Cytoskeletal structural protein	DQ485686	EB031070
GAPDH1	Glyceraldehyde-3-phosphate dehydrogenase	Glycolytic enzyme	AB300322	EB032115
GAPDH2	Glyceraldehyde-3-phosphate dehydrogenase	Glycolytic enzyme	AB291587	--
ANXA2	Annexin A2	Membrane bridging	AB3605934	EB030312
GST	Glutathione S-transferase	Detoxification	AB360595	EB034437
ODC	Ornithine decarboxylase	Polyamine synthesis	--	EB039871
HPRT1	Hypoxantine phosphoribosyl transferase	Purine nucleotide synthesis	AB360596	EB038066
UBQ	Ubiquitin	Protein degradation	AB291588	EB038831
eEF1A1	Elongation factor 1 alpha	Protein synthesis	AB326302	EB034150
RPL13a*	60S ribosomal protein L13a	Structural component of the large 60S ribosomal subunit	AB360598	EB030954
RPS4	40S ribosomal protein S4	Structural component of the small 40S ribosomal subunit	AB291557	EB034819
18S rRNA	18S ribosomal RNA	Ribosome structure	AM882675	CN655651

*: Genes are paralogous in the two flatfishes.

--: Gene expression was not tested.

### RNA isolation and gene expression analysis

Homogenization of Senegalese sole larvae was carried out using Lysing Matrix D (Q-BioGene Inc., Carlsbad, CA, USA) for 40 s at speed setting 6 in the Fastprep FG120 instrument (Bio101, Thermo Savant Instruments). Total RNA was isolated from 50 mg of pooled larvae using the RNeasy Mini Kit (Qiagen, Valencia, CA, USA). For Atlantic halibut larvae, RNeasy Fibrous Tissue Mini Kit (Qiagen) was used to isolate total RNA. Fish were transferred into a 1.5 ml microfuge tube with RLT buffer from the kit and disrupted with a pellet pestle (Kimble-Kontes, Vineland, NJ). The mixture was passed through 18- and 21-gauge needles, to homogenize the sample and to reduce the viscosity. All RNA isolation procedures were performed in accordance with the manufacturer's protocol. In all cases, total RNA was treated twice with DNase I using the RNase-Free DNase kit (Qiagen) for 30 min for Senegalese sole or DNA-free (Ambion, Austin, TX, USA) for Atlantic halibut in order to avoid amplification of genomic DNA. For Senegalese sole RNA, sample quality was checked using Experion (Bio-Rad) and quantification was performed spectrophotometrically. Total RNA (1 μg) from each sample was reverse-transcribed using the iScript™ cDNA Synthesis kit (Bio-Rad, Hercules, CA, USA). Lack of genomic DNA contamination was confirmed by PCR amplification of RNA samples in the absence of cDNA synthesis.

Real-time analysis was carried out on an iCycler (Bio-Rad). Reactions were performed in a 25 μL volume containing cDNA generated from 10 ng of original RNA template, 300 nM each of specific forward (F) and reverse (R) primers (Table 3), and 12.5 μl of iQ™ SYBR Green Supermix (Bio-Rad). Matching oligonucleotide primers were designed using Oligo *v*6.89 software (Medprobe, Oslo, Norway) or primer 3 (Whitehead Institute for Biomedical Research). The amplification protocol used was as follows: initial 7 min denaturation and enzyme activation at 95°C, 40 cycles of 95°C for 15 s and 70°C for 30 s, and for Atlantic halibut, initial 7 min denaturation and enzyme activation at 95°C, 40 cycles of 95°C for 20 s, 58°C for 20 s and 72°C for 20 s. Each assay was performed in duplicate. To estimate efficiencies, a standard curve was generated for each primer pair based on known quantities of cDNA (10-fold serial dilutions corresponding to cDNA transcribed from 100 to 0.01 ng of total RNA). All calibration curves exhibited correlation coefficients higher than 0.99 and the corresponding real-time PCR efficiencies were in the range 0.86–1.01 (Table 3).

**Table 3:** Primer sequences for candidate normalization genes. Amplicon size and efficiency (E) in Senegalese sole and Atlantic halibut are indicated.

Gene	*S. senegalensis*			*H. hippoglossus*		
		
	Primer Sequence (5'→3')	Amplicon length	E	Primer Sequence (5'→3')	Amplicon length	E
	
ACTB1	CTCCATCGTCCACCGCAAGTGCTTC (F)	92 bp	0.90	AAGAAGAAAGAAAAGACGGAGGA	81 bp	0.98
	TGTCCATTCGTGCAAGATCGGGAGA (R)			AAGATTTGCCAGAGATGTTTCC		
ACTB2	AATCGTGACCTCTGCTTCCCCCTGT	113 bp	0.96	GGTGTATGCTGAGCCCAAGA	114 bp	0.98
	TCTGGCACCCCATGTTACCCCATC			CCTGGGACGCTCTGTACTTC		
GAPDH1	TGTTCGTCTGGAGAAACCCGCCAAA	160 bp	0.94	GTGTCAGTGGTTGACCTGA	212 bp	0.99
	AGCGCCAGCATCAAAGATGGAGGAG			AGCTTGACAAAGTGGTCATT		
GAPDH2	AGCCACCGTGTCGCCGACCT	107 bp	0.95			
	AAAAGAGGAGATGGTGGGGGGTGGT					
ANXA2	GGTCAACTTCGCAGCGGGAAACCA	69 bp	0.94	CGACTCGCCAAGAAGGATCT	129 bp	0.99
	CCATGAAGGCTAAAGGGGCAAAGGA			CCCTTCATGGAAGCTTTGAG		
GST	TTTCCAACCATCGCTTATGTCTTCCGTTT	109 bp	0.86	AAGAACCTGCAGGGCTACAA	135 bp	0.99
	AGCTTTTTTTGATACTGGGCCGGTCCT			CCCATGTTTGAAGGAAGGAA		
ODC				GCTTCTGCTTACACGCTAGT	143 bp	1.01
				TTGAAAGATCCGTACACTCC		
HPRT1	GGACATGGCGACATCCAGCTCCTGTGTT	120 pb	0.96	GGGGTATGACCTGGACCTTT	108 bp	0.99
	TGGGGGATGTAGACCCTCTCCAGGTCAG			TTGGCCAGTCTCTCTGTCCT		
UBQ	AGCTGGCCCAGAAATATAACTGCGACA	93 bp	0.95	ATCGAGAATGTCAAGGCTAA	140 bp	0.98
	ACTTCTTCTTGCGGCAGTTGACAGCAC			AGATGCAGAGTGGATTCTTT		
eEF1A1	GATTGACCGTCGTTCTGGCAAGAAGC	142 bp	0.95	AAGAGGACCATCGAGAAGTT	141 bp	0.97
	GGCAAAGCGACCAAGGGGAGCAT			GTCTCAAACTTCCACAGAGC		
RPL13a	GCTGCGCTGGAGAGGCTGAAGGTGT	115 bp	0.97	CGGTTCAATAAGGTTCTGCT	111 bp	0.99
	CTCAAGAAAATACCACAGGATGGGCTTCAA			CATCTCACAACCACCACTTT		
RPS4	GTGAAGAAGCTCCTTGTCGGCACCA	83 bp	0.94	GCCAAGTACAAGCTGTGCAA	138 bp	1.00
	AGGGGGTCGGGGTAGCGGATG			AGGTCGATCTTGACGGTGTC		
18S rRNA	GAATTGACGGAAGGGCACCACCAG	148 bp	0.97	GGGAGGTAGTGACGAAAAAT	148 bp	1.00
	ACTAAGAACGGCCATGCACCACCAC			AAGATACGCTATTGGAGCTG		

In order to ensure comparability among data, the threshold values among plates were normalized by manually setting the Ct values corresponding to at least two previous reference samples. Raw Ct values were transformed to quantities using the comparative Ct method and specific efficiencies [37]. Data obtained were converted into correct input files, according to the requirements of the software, and analyzed using geNorm [20], and NormFinder [21].

## Authors' contributions

CI carried out the gene expression analysis in Senegalese sole and helped to draft the manuscript MPM carried out the gene expression analysis in halibut, carried out sequence analyses and helped to draft the manuscript. EA performed the Senegalese sole cultures and samplings. JPC participated in the study design and helped to draft the manuscript. MR participated in the study design and drafted the manuscript. MM designed the study, carried out sequence analyses, and drafted the manuscript. All authors read and approved the final manuscript.
